# Biocompatible hydrogels for the controlled delivery of anti-hypertensive agent: development, characterization and *in vitro* evaluation

**DOI:** 10.1080/15685551.2018.1445416

**Published:** 2018-03-12

**Authors:** Shahid Nawaz, Samiullah Khan, Umar Farooq, Malik Salman Haider, Nazar Muhammad Ranjha, Akhtar Rasul, Ahmad Nawaz, Numera Arshad, Rabia Hameed

**Affiliations:** ^a^ Faculty of Pharmacy, Bahauddin Zakariya University , Multan, Pakistan; ^b^ Faculty of Pharmacy and Alternative Medicine, The Islamia University of Bahawalpur , Punjab, Pakistan; ^c^ Department of Pharmacy, Government College University Faisalabad , Faisalabad, Pakistan; ^d^ Department of Pharmacy, COMSATS Institute of Information Technology , Abbottabad, Pakistan; ^e^ Faculty of Pharmacy, Punjab University Lahore , Lahore, Pakistan

**Keywords:** Grafting polymerization, anti-hypertensive agents, controlled release, pH sensitivity, porous gels

## Abstract

The aim of the present exploration was to develop novel pH-sensitive cross-linked Gelatin/Polyvinyl pyrrolidone hydrogels using different ratios of both the polymers and to investigate the effect of polymers and degree of crosslinking on dynamic, equilibrium swelling and *invitro* release pattern of the model drug (captopril). Grafting polymerization technique was used for the preparation of these hydrogels using glutaraldehyde as crosslinking agent. These polymeric materials were then used as model systems to envisage various important characterizations like FTIR (Fourier transform infrared spectroscopy), XRD (X-ray diffraction) and scanning electron microscopy (SEM). Phosphate buffers of pH 1.2, 6.5 and 7.5 were used for swelling and *invitro* drug release profile investigation. Different parameters like swelling analysis, porosity, sol-gel analysis, average molecular weight between crosslinks (Mc), solvent interaction parameter (χ), volume fraction of polymer (V_2,s_) and diffusion coefficient that affects the drug release behavior were also determined. Higher swelling and release was observed at lower pH values. FTIR spectra showed interaction between gelatin and polyvinyl pyrrolidone and successful formation of cross-linked structure. Pulsatile drug release study showed the controlled delivery of model drug. The release of drug occurred through non-fickian diffusion or anomalous mechanism. Aforementioned characterizations reveal successful formation of copolymer. pH sensitive swelling ability and drug release behavior suggest that the rate of polymer chain relaxation and the rate of drug diffusion from these hydrogels are comparable which also predicts their possible use for site specific captopril delivery.

## 1. Introduction

Hydrogels refers to 3-dimensional hydrophilic polymeric networks, having numerous biomedical application. These polymeric networks are of great importance to biomaterial sciences due to their hydrophilic properties and biocompatible potential. Being flexible and special soft, these polymeric material can absorb sufficient water, saline or physiological fluids and have the ability to retain it. Although they are insoluble in water at normal body temperature and pH, yet they absorb sufficient water to swell. [1–7]

Hydrogels as biomaterials has a range of medical applications like artificial implantation [8], artificial skin development [9], swelling controlled drug delivery system [10], wound dressing and humidity sensor. [11] Hydrogel are also termed as smart or intelligent gels. [12] The characteristics of smart gels are to receive, transmit, and mainly responds to external stimuli. [13] These stimuli include temperature, pH, electric current and ionic strength. [14] The response of hydrogel to environmental stimuli is of great importance from medical and biological point of view. Human body parts have different pH values. [15] Hydrogels can easily be washed to remove uncross-linked polymers or un-reacted contents due to their swelling characteristics. The water content in hydrogel makes them a very distinguish class. Hydrogel, having good mechanical strength, can be obtained by the preparation of interpenetrating polymer network (IPN). [16]

Hydrogels can be prepared via physical interactions such hydrogen bonding, hydrophobic interactions, host-guest molecules interactions etc. or they can prepared through chemical crosslinking via creating permeant bonds.

Chemically cross-linked gel can be prepared by various methods such as radical polymerization or by using cross-linking agent (e.g., glutaraldehyde, epichlorhydrin) or by inducing radiation (e.g., UV radiation, gamma radiation). The strength and swelling of hydrogels can be adjusted or controlled by the quantity of cross-linking agent. Environmentally responsive gels can also be fabricated upon the addition of specific type of monomers [17–19]. Figure 1 indicates the graphical representation of different crosslinking methods.

**Figure 1.**
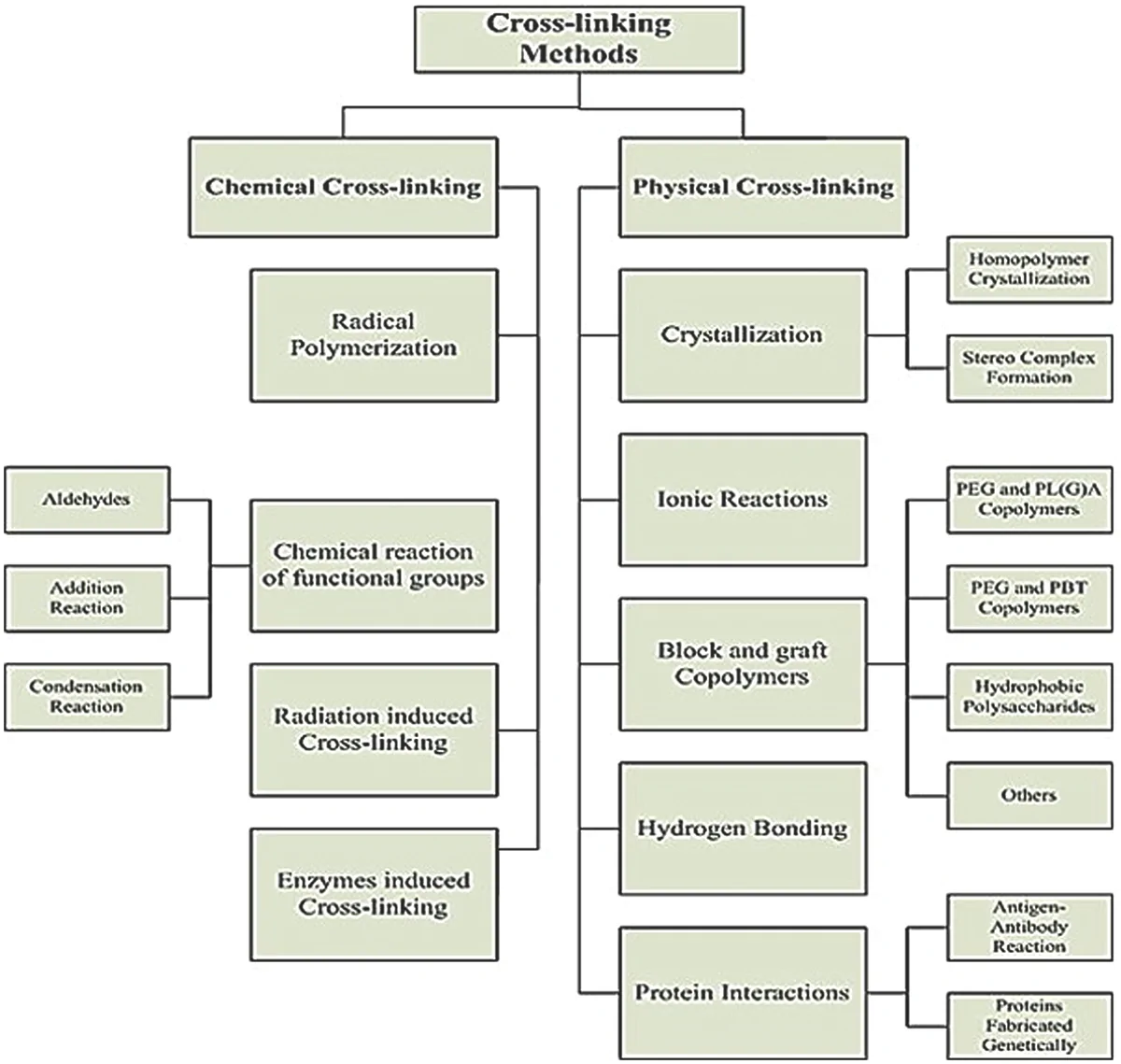
The graphical representation of different crosslinking methods.

Gelatin, a naturally occurring polypeptide, is obtained from collagen through hydrolysis, which is obtained from animal sources (porcine, bovine or fish collagen), bone and tissues (connective tissues) [20,21]. It has different sequence of amino acids [22]. Various products are obtained from gelatin that are medically important due to their safety, non-toxic, non-carcinogenic and non-immunogenic nature and its biocompatibility [23]. At 40 °C, gelatin solution remains in the form of solution, and on lowering the temperature, it forms thermos- reversible gel [24].

Polyvinyl pyrrolidone (PVP) is a synthetic polymer of great importance and is freely soluble in water, biocompatible, chemically stable and has the film forming property [25,26]. It has many uses in the fields of cosmetics, medicines, adhesives, food stuffs, photo sensitive materials and paints. [27–29] Using N-vinyl pyrrolidone as raw material, it can be prepared either by bulk polymerization [30] or by solution polymerization [31].

Captopril (1-[(2S)-3-mercapto-2-methylpropionyl]-L-proline) belongs to the class of drugs known as angiotensin converting enzyme inhibitors and is given orally. The mode of action of captopril is yet not clearly understood. It causes suppression of the renin-angiotensin-aldosterone system and ultimately has benefits in hypertension and heart failure. Captopril inhibits angiotensin converting enzyme (ACE) peptidyl dipeptide carboxy hydrolase which converts angiotensin I into II. It has a shorter half-life of 2 h and rapidly absorbed after oral administration with peak blood levels of approximately 1 mcg/l after a 100 mg dose within 30 min to 1 h. Figure 2 indicates the chemical structure of captopril.

**Figure 2.**
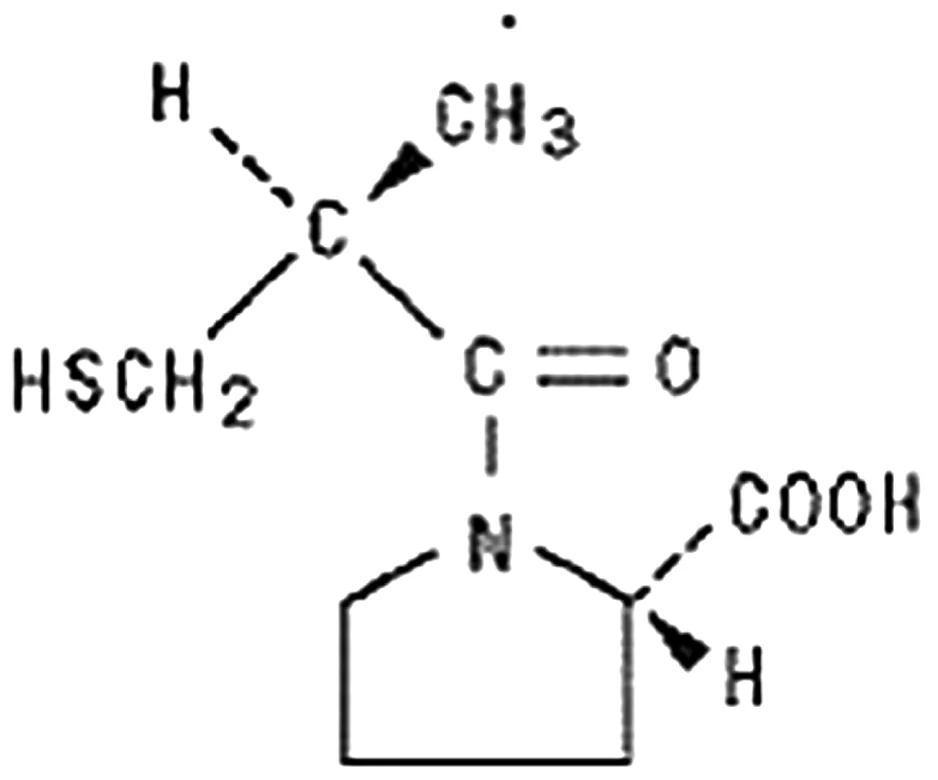
Chemical structure of captopril.

In current investigation an attempt has been made to develop novel biocompatible and biodegradable hydrogels for the controlled delivery of captopril in order to address the silent disease of hypertension. The prepared hydrogel samples were developed to obtain the following objectives; (1) To develop different hydrogel samples with different feed composition ratios and degree of crosslinking (2) To perform swelling experiments in order to investigate the effect of composition and crosslinking ratio in phosphate buffer solutions of variable pH values (3) To investigate the effect of pH and composition on release of captopril in phosphate buffer solutions of variable pH values and to confirm the controlled delivery of model drug (4) To evaluate sol-gel fraction analysis, porosity measurement, networking parameters and diffusion coefficient. (5) To evaluate the best release mechanism by applying various mathematical release models. (6) To confirm the network structure of hydrogels by various characterization tools like Fourier transform infrared (FTIR) spectroscopy, X-ray diffraction (XRD) and scanning electron microscopy (SEM) was used to look into the morphology of the hydrogel samples respectively.

## 2. Experimental

### 2.1. Materials

Gelatin (Ge) (MW ~ 402.47 g/mol) (Merck Germany) and Polyvinyl pyrrolidone (Mw~40,000; Biomedical, France) were used as polymers. Glutaraldehyde (GA) (Scharlu Chemie S.A.) was used as crosslinker. Captopril was gifted by GSK and was used as model drug. Potassium bromide (KBr) FTIR’s grade was bought from Fischer scientific (United Kingdom). Potassium dihydrogen phosphate (AppliChem, Darmstadt Germany), hydrochloric acid (Merck Germany), sodium hydroxide (Merck, Darmstadt Germany), acetic acid and sodium chloride were also used and all these chemicals were of analytical grade.

### 2.2. Synthesis of gelatin and polyvinyl pyrrolidone (Ge/PVP) hydrogels

In present work a series of cross-linked hydrogels based on gelatin and polyvinyl pyrrolidone with different ratios of polymers and crosslinking agent were prepared by method reported earlier [32]. Table 1 indicates the feed ratio compositions of Ge/PVP hydrogels.

**Table 1.** Feed composition of different formulations of Ge/PVP based hydrogels.

Sample codes	Ge/100 g solution	PVP/100 g solution	Ge/PVP (Wt %)	GA/100 g of Ge/PVP (Wt. %)	GA/100 g solution
S_1_	14.5	3.0	82.85/17.15	3.8	2.660
S_2_	16.5	3.0	84.61/15.39	3.8	2.964
S_3_	19.0	3.0	86.36/13.46	3.8	3.344
S_4_	16.5	3.5	82.50/17.50	3.8	3.040
S_5_	16.5	4.0	80.48/19.52	3.8	3.116
S_6_	16.5	4.5	78.57/21.43	3.8	3.192
S_7_	16.5	3.0	84.61/15.39	3.5	2.730
S_8_	16.5	3.0	84.61/15.39	4.0	3.120
S_9_	16.5	3.0	84.61/15.39	4.2	3.276

Briefly, calculated amount of Ge was dissolved in predetermined amount of 2% v/v solution of glacial acetic acid with continuous stirring at 250–300 rpm at 60 ± 0.5 °C for 90 min. Weighed amount of PVP was dissolved in small quantity of distilled water under continuous stirring of 400–450 rpm. Both the polymer solution were mixed together and continued stirring for 90 min for complete mixing. The final volume make up was made with the addition of sufficient distilled water to 100 g. Calculated amount of crosslinker was added to the polymer mixture and stirred for 30 min at 250 rpm. The final co-polymer mixture was poured into the glass tubes and tubes were deoxygenated with nitrogen gas bubbling. The deoxygenated glass tubes were fitted with the lid and placed to dry at room temperature for 72 h. The hydrogels of Ge/PVP were cut into cylindrical discs of 5 mm. The hydrogel discs were placed at room temperature in petri dishes for drying. The un-crosslinked polymer was removed by placing the dried discs in washing medium (distilled water). The hydrogel discs were placed in the washing medium and the washing medium was changed on daily basis until the pH of the old medium was found similar to that of the fresh medium. After the discs were washed, they were placed in vacuum oven until their weight becomes constant at a temperature of 40–45 °C. Figure 3 refers to the presumptive chemical crosslinking between Ge and PVP in the presence of glutaraldehyde.

**Figure 3.**
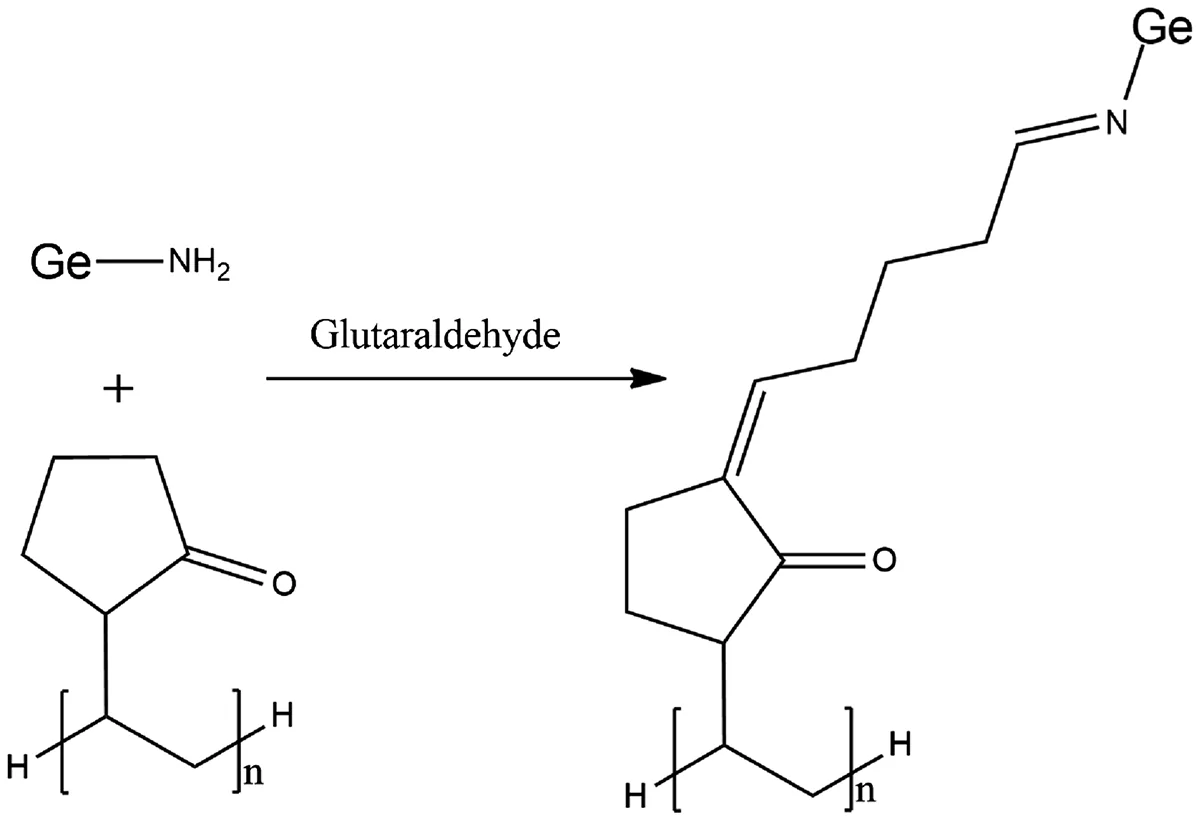
Presumptive chemical structure of Ge/PVP based hydrogels.

### 2.3. Characterization of Ge/PVP hydrogels

#### 2.3.1. Swelling experiments

Degree of swelling can be described as water absorptivity of the hydrogel. Fresh 0.05 M phosphate buffer solutions of different pH i.e., 1.2, 5.5, 6.5 and 7.5 were used for the determination of dynamic and equilibrium swelling coefficient. Dried discs were weighed, immersed in a solution of desired pH and left to swell at temperature of 37 °C. For dynamic swelling, the discs were taken out of the buffer solution with specific period of time, blobbed, and placed in the same buffer solution after weighing. Following equation was used to calculate the swelling coefficient for each sample [32].

(1) $$q=\frac{W_{h}}{W_{d}}$$

where *q* is the swelling coefficient, *W*_*h*_ is the weight of hydrogel disc in swollen form and *W*_*d*_ is the weight of the hydrogel disc in dry form.

#### 2.3.2. Equilibrium swelling ratio

For investigation of equilibrium swelling the discs were remained in the same buffer solution, weighed thoroughly until the weight became constant. During equilibrium swelling investigation, the discs were weighed at regular interval of time. Following equation was used to calculate equilibrium swelling [32,33].

(2) $$S_{\left(Eq\right)}=\frac{W_{h}}{W_{d}}$$

*W*_*h*_ and *W*_*d*_ denotes the weight of hydrogel disc in swollen and dried form respectively.

### 2.4. Sol-gel fraction analysis

Sol part refers to the unreacted portion of formulation, while gel part indicates the part of the polymeric mixture which polymerized and converted to gel form. Sol-gel analysis is used for the determination of gel fraction. For the determination of gel fraction, extraction was carried out in soxhelt apparatus using deionized water as extraction medium. Pre-weighed dried discs were placed in soxhelt apparatus at 100 °C for four hours. These discs were then dried first at room temperature and then in oven at 45 °C till their weight becomes constant. Gel fraction was calculated as follows. [33]

(3) $$\text{Sol}\;\text{fraction}\left(\%\right)=\left[\frac{W_{o}-W_{i}}{W_{o}}\right]\;\times \;100$$

(4) $$\text{Gel}\;\text{fraction}\left(\%\right)\;=100-\text{Sol}\;\text{fraction}$$

where *W*_0_ is the weight of hydrogel disc before extraction and *W*_*i*_ is the weight of hydrogel disc after extraction.

### 2.5. Porosity

Solvent replacement method was used for porosity measurement. Weighed discs of the hydrogel were placed for overnight in pure ethanol. After estimated time, discs were removed of the ethanol and blobbed with blotting paper to remove extra ethanol present on the surface of the discs and weighed again. Equation used for calculating % porosity is given as follows [33].

(5) $$\text{Porosity}=\left[\frac{M_{2}-M_{1}}{\mathrm{pV}}\right]\;\times \;100$$

where *M*_1_ and *M*_2_ represents weights of the hydrogel discs prior to and after placing in absolute ethanol respectively. *P* is ethanol’s density and *v* is hydrogel volume.

### 2.6. Networking parameters of Ge/PVP hydrogel

#### 2.6.1. Diffusion coefficient

Diffusion coefficient is actually a factor of proportionality representing the amount of substance diffusing across a unit area through a unit concentration gradient in unit time. Equation used for the calculation of diffusion coefficient is given below. [32]

(6) $$D\;=\;\pi {\left[\frac{h.\theta }{4.Q_{\mathrm{eq}}}\right]}^{2}$$

where *D* indicates the diffusion coefficient of the hydrogels, *Q*_*eq*_ represents the equilibrium swelling of the gel, ө is the slop of the linear part of the swelling curves and h shows the initial thickness of sample before subjecting to swelling experiment.

#### 2.6.2. Average molecular weight between crosslinks (M_c_)

Degree of crosslinking in the hydrogel network is represented by the average molecular weight between two adjacent crosslinks. It describes the mixing phenomenon between the polymer and the liquid. *M*_*c*_ was determined by Flory-Rehner equation. [33]

(7) Mc=-dpvsv2,s1/3-v2,s2ln1-v2,s+v2,s+xv2,s2

where *d*_*p*_ and *d*_*s*_ represent the densities of polymer and solvent respectively, *v*_*s*_ is the molar volume of the solvent, *χ* is the Flory parameter- solvent interaction parameter, *V*_2,*s*_ is the volume fraction of the polymer in the swollen state.

*V*_2*,s*_ and *χ* are calculated as follows. [33]

(8) $$\chi =\frac{\ln\left(1-\nu_{2,s}\right)+\nu_{2,s}}{\nu_{2,s}^{2}}$$

(9) $$V_{2,s}={\left[1+\frac{d_{p}}{d_{s}}\left(\frac{M_{a}}{M_{b}}-1\right)\right]}^{-1}$$

where *M*_*b*_ and *M*_*a*_ are the masses of dry and swollen polymers respectively.

#### 2.6.3. Cross-linked density (q)

Cross-linked density (*q*) can be determined from average molecular weight between crosslinks (*M*_*c*_) by using following equation. [33]

(10) $$q=\frac{M_{C}}{M_{r}}$$

where *M*_*r*_ represents the molar mass of the repeating units and is calculated as

(11) $$M_{r}=\frac{m\text{Ge}M\text{Ge}+m\text{PVPMPVP}+m\text{GAMGA}}{m\text{Ge}+m\text{PVP}+m\text{GA}}$$

where *m*_Ge_, *m*_PVP_ and *m*_GA_ represents the feed masses of gelatin, poly vinyl pyrrolidone and glutaraldehyde respectively. While *M*_Ge_, *M*_PVP_ and *M*_GA_ represents the molar masses of gelatin, poly vinyl pyrrolidone and glutaraldehyde respectively used in the synthesis of hydrogel.

### 2.7. Drug loading and release

#### 2.7.1. Drug loading method and its determination

Different samples were selected for drug loading as well as release studies. Six samples were pointed out of which three have different concentration of gelatin and three have varying concentration of crosslinking agent i.e., 3.5, 3.8 and 4.0%. This selection of samples for release and loading studies were based on their maximum equilibrium swelling. Double distilled water was used to prepare 1% solution of the drug. For drug loading into the hydrogel discs, the dried discs were first weighed in then soaked in the prepare drug solution up to their equilibrium swelling. After achieving the equilibrium state, the hydrogel discs loaded with drug were allowed to dry at 25 °Cand then at 45 °Cin oven till the attainment of constant weight. [34]

Two different methods were used for determination of % drug loading in the hydrogel discs.

In first method called weight method, also called dry weight method, loaded drug is calculated by the following formula.

(12) $$\text{Amount}\;\text{of}\;\text{drug}\;=\;W_{D}-W_{d}$$

Percent drug loading was calculated by the following formula.

(13) $$\text{Drug}\;\text{loading}\left(\%\right)\;=\;\left[{\frac{\left(W_{D}-W_{d}\right)}{W}}_{d}\right]\times 100$$

*W*_*d*_ = weight of unloaded discs in dried form

*W*_*D*_ = weight of the drug loaded discs in dried form

In second method called extraction method, drug loading was determined by continuous extraction of drug from the gels and replacing the sample on daily basis, till extraction and calculation of all the drug. This method employed 25 ml of fresh double distill water for extraction of drug from the disc till the solution was free of drug. Spectrophotometry was employed for determination of concentration of drug in the solution. The total amount of loaded drug was determined by summing up the drug portion present in each portion of the extract solution.

#### 2.7.2. Drug release study

Drug release mechanism was investigated at 37 °C using USP type II apparatus. The drug loaded hydrogel discs were placed in dissolution apparatus containing dissolution medium at 37 °Cand stirring was done at 100 rpm for obtaining uniform concentration of the drug in the medium. Freshly prepared phosphate buffer were used as dissolution medium. 900 ml of medium was used for each sample. Amount of drug release at time (t) was obtained spectrophotometrically at 227 nm. Each time 5 ml solution was drawn for UV analysis and drawn amount is compensated by fresh USP phosphate buffer solution [33,34].

#### 2.7.3. Release pattern analysis

The drug loaded hydrogel discs were analyzed for drug release pattern. Based on the relative rate of diffusion and swelling of the polymers drug release profiles from the Ge/PVP hydrogels have been studied by Zero order, First order, Higuchi and Korsmeyer-Peppas models. [35]

The pH of the dissolution medium was used as function for the release of drug from different samples with different polymeric compositions.

Semi-empirical power formula, given by Pappas, was used to have an insight into the release mechanism. Following models were used for drug calculation.

(14) $$\text{Zero}-\text{order}\;\text{kinetics}:F_{t}=K_{o}t$$

where *F* is used to denote the fractional release of the drug in time *t* and *K*_0_ represents the constant for zero order release [35]

(15) $$\text{First}-\text{order}\;\text{kinetics}:\text{In}\left(1-F\right)\;=\;-K_{1}t$$

where *F* represents the proportion of drug release in time *t* and *K*_1_ is used to denote the constant for first order release [36]

(16) $$\text{Higuchi}\;\text{model}:F=K_{2}t1\;/\;2$$

where *K*_2_ denotes Higuchi constant and F is the proportional drug release in time *t* [37].

(17) $$\text{Korsmeyer}-\text{Peppas model}:M_{t}/M\mu =K_{3}t^{n}$$

where *M*_*t*_ represents the absorb amount of water at any time *t*; *M*_*∞*_ is the equilibrium mass uptake or absorb amount of water in equilibrium state; *K*_3_ is constant and n represents exponent of swelling mechanism. For the value of *n* = 0.45, release order is considered to be Fickian and when it is greater than 0.45 and less than 1, then release is considered to be non-Fickian diffusional mechanism. In order to investigate the kinetic data or n values, the values of drug release and swelling co-efficient should be significant [38].

### 2.8. Instrumental analysis

#### 2.8.1. Fourier transformed infrared spectroscopy

The dried discs of Ge/PVP hydrogel were crushed into powder using pestle and mortar. Potassium bromide was mixed in 1:100 proportion with crushed powdered hydrogel material, and placed in oven at 40 °Cfor drying. Semitransparent discs with 12 mm diameter were prepared by applying the pressure of 65 KN (pressure gauge, schimadzu) for 2 min. Using the wavelength range of 4000–500 cm^−1^, the FTIR spectrum was recorded from FTIR spectrometer (FR-IT 8400 S, Shimadzu) [32].

#### 2.8.2. X-ray Diffraction analysis

Bruker D8 Discover (Germany) instrument was used for the analysis of X-ray diffraction (XRD) patterns were recorded for drug loaded and unloaded hydrogels. The instrument was operated at at 25 °C temperature, using the nickel filtered CuKα radiation (*λ* = 1.54050° A) and operating at voltage (40 kV), and current (35 mA), step scan 0.1° of 2*θ* and 1 s of counting time. Eva software was used for the data processing (Evaluation Package Bruker, Germany). The range of diffraction angle was 10°**–**70° at 2*θ* [32].

#### 2.8.3. Scanning electron microscopy (SEM)

Surface and cross-sectional morphology of unloaded IPN samples and drug-loaded samples were investigated using SEM (440, Leica Cambridge Ltd., Cambridge, UK). Hydrogel samples were mounted on an aluminium mount and sputtered with gold palladium. The specimen mounts were then coated with 60% gold and 40% palladium for 30s with 45 mA current in a sputter coater (Desk II, Denton Vacuum, Moorestown, NJ, USA). The coated specimens were then observed on SEM using an accelerating voltage of 20 kV at a tilt angle of 30^**◦**^ to observe the microstructure of the sample [33].

### 2.9. Statistical analysis

For the statistical analysis of data, Student’s *t*-test has been applied to compare the results and to determine the statistical significant/ non-significant interpretation at 95% confidence interval, *p*-value less than 0.05 was considered as significant difference in results.

## 3. Results and discussion

### 3.1. Effect of pH on swelling and on drug release of Ge/PVP hydrogel

The swelling behavior of the hydrogel can be determined, when the pH of the swelling medium, pk_a_ and pk_b_ of the acidic and basic components of the hydrogel polymers are known.

For determination of pH dependent swelling of Ge/PVP hydrogel, the hydrogel discs of different formulation were placed in swelling medium of various pH 1.2, 5.5, 6.5 and 7.5. As both carboxylic and amino groups are present in the formulation, so maximum swelling was observed at pH 1.2 followed by pH 7.5. When *pk*_*b*_ value of the basic component is greater and *pk*_*a*_ value of the acidic component is lower than the pH of the swelling medium, it results in deprotonation or ionization of both the components. As a result of greater *pk*_*b*_ value than the pH of the swelling medium at pH 1.2, maximum swelling was observed at this pH. At pH 1.2 –NH^+^ ions are formed and these cations have electrostatic repulsive forces which causes the swelling of the hydrogel. Above the *pk*_*a*_ value, –COOH group present in Ge deprotonated to COO^−^. Thus at pH 7.5 there also occur electrostatic forces of repulsion between the anions formed and results in the swelling of hydrogel. Figure 4 indicates the schematic representation of acid base reactions in corresponding buffer media.

**Figure 4.**
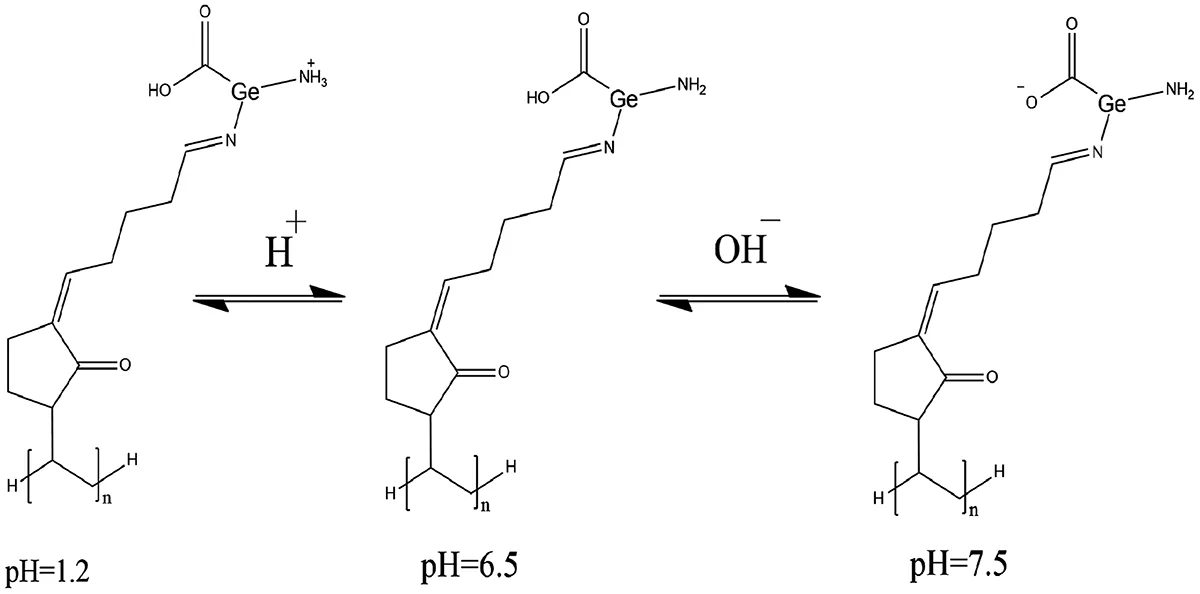
The schematic representation of acid base reactions in corresponding buffer media.

Drug release studies were carried out on samples that showed maximum swelling characteristics. Three samples (S_1_, S_2_ and S_3_) with different gelatin concentration and three samples with different glutaraldehyde concentration (S_7_, S8 and S_9_) were loaded with the model drug. Table 2 and 3 indicates the amount of loaded and released drug from selected samples.

**Table 2.** Amount of captopril loaded in different samples of GE/PVP hydrogels.

Sample codes	Amount of captopril loaded (g/g of dry gel)	
	By swelling	By extraction
S_4_	0.0562	0.0490
S_5_	0.0611	0.060
S_6_	0.0694	0.672
S_7_	0.0546	0.0517
S_8_	0.0518	0.0462
S_9_	0.0492	0.0424

**Table 3.** % Release of captopril from different samples of Ge/PVP hydrogels.

Sample codes	pH 1.2	pH 6.5	pH 7.5
S_1_	75.31	52.37	61.72
S_2_	76.57	53.21	59.16
S_3_	79.12	54.03	64.97
S_7_	77.42	56.24	65.54
S_8_	73.65	49.42	59.63
S_9_	72.24	47.05	58.82

Captopril was employed as model drug. Phosphate buffer solution of different pH values (7.5, 6.5 and 1.2) were used for the determination of drug release behavior. It was noted that drug release was maximum at 1.2 followed by highest release at 7.2 pH as shown in the Table 3. The swelling behavior can be correlated with drug release as maximum swelling of Ge/PVP hydrogel was also observed at acidic pH.

### 3.2. Effect of Ge concentration on swelling and on drug release of Ge/PVP hydrogel

Three formulation of Ge/PVP hydrogel with increasing concentration of Ge as 14.5, 16.5 and 19.0 g keeping the concentration of PVP and GA constant were used to investigate their swelling behavior in solutions of different pH. Increase in the swelling co-efficient was observed as Ge concentration increases. Protonated -NH^+^ ions formed at pH lower than the pk_b_ value of the Ge chains, thus cationic repulsive forces between the NH^+^ ions are the main cause of higher swelling at lower pH. Saarai *et al* *.* fabricated hydrogels based on Ge/Na alginate and found the same results. [39] Figure 5 shows the effect of increasing Ge concentration on equilibrium swelling degree of Ge/PVP hydrogels.

**Figure 5.**
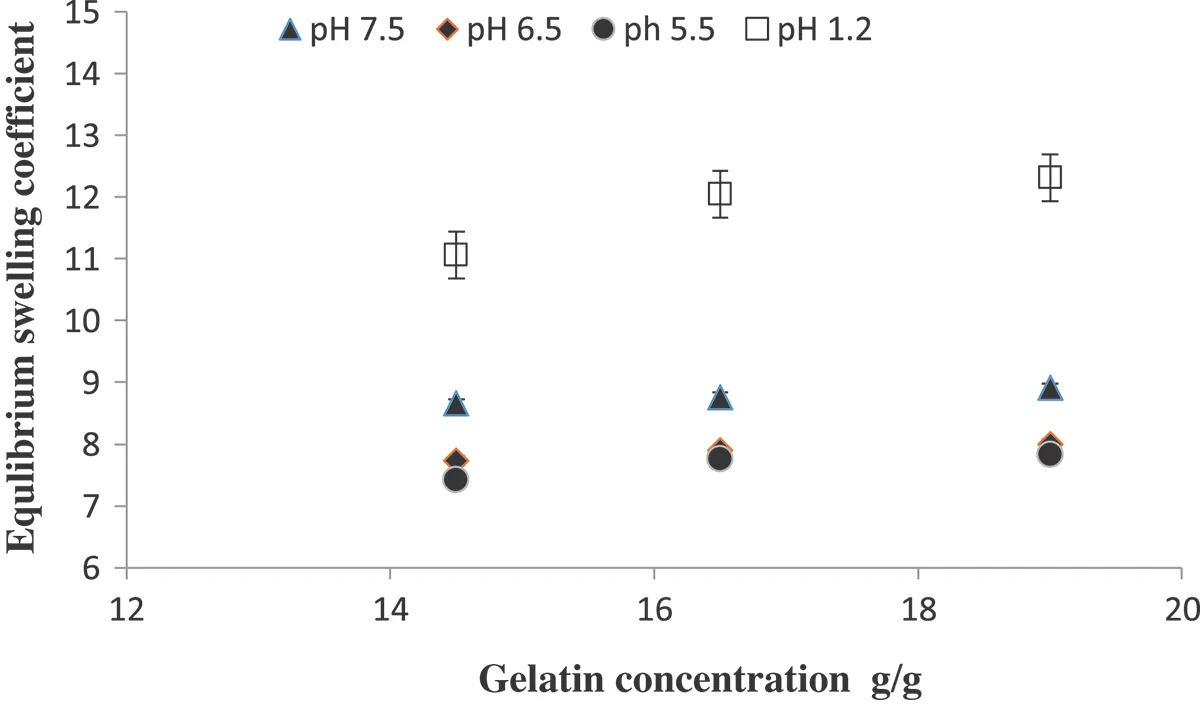
Equilibrium swelling ratio of Ge/PVP hydrogels with different concentrations of Ge using glutaraldehyde (GA) as crosslinking agent in solutions of different pH; pH 1.2 (□), pH 5.5 (●), pH 6.5 (♦) and pH 7.5 (▲).The data present the mean ± standard deviation of *n* = 3 individual readings.

Drug release studies of captopril were carried out in different pH solutions (1.2, 6.5, and 7.5) for samples S1, S2, S3 having 14.5, 16.5 and 19.0 g of Ge respectively. Figure 8(A) present the data showing the effect of Ge on percent release of captopril from loaded hydrogel for selected samples. It was observed that Ge concentration and drug release were proportional to each other i.e., increase in Ge concentration results in increased release of the drug.

### 3.3. Effect of PVP concentration on swelling and drug release of Ge/PVP hydrogels

In order to investigate the effect of PVP, three different formulation with varying concentration of PVP as 3.5, 4.0 and 4.5 g keeping Ge and GA constant (3.8 wt. % of Ge and PVP) were prepared and investigated for their swelling behavior in solutions of different pH. It was observed that increase in the concentration of PVP in the formulation also causes increase in the swelling ratio of the hydrogel. Figure 6 illustrate the effect of PVP concentration on equilibrium swelling coefficient of Ge/PVP hydrogels in different buffer solutions.

**Figure 6.**
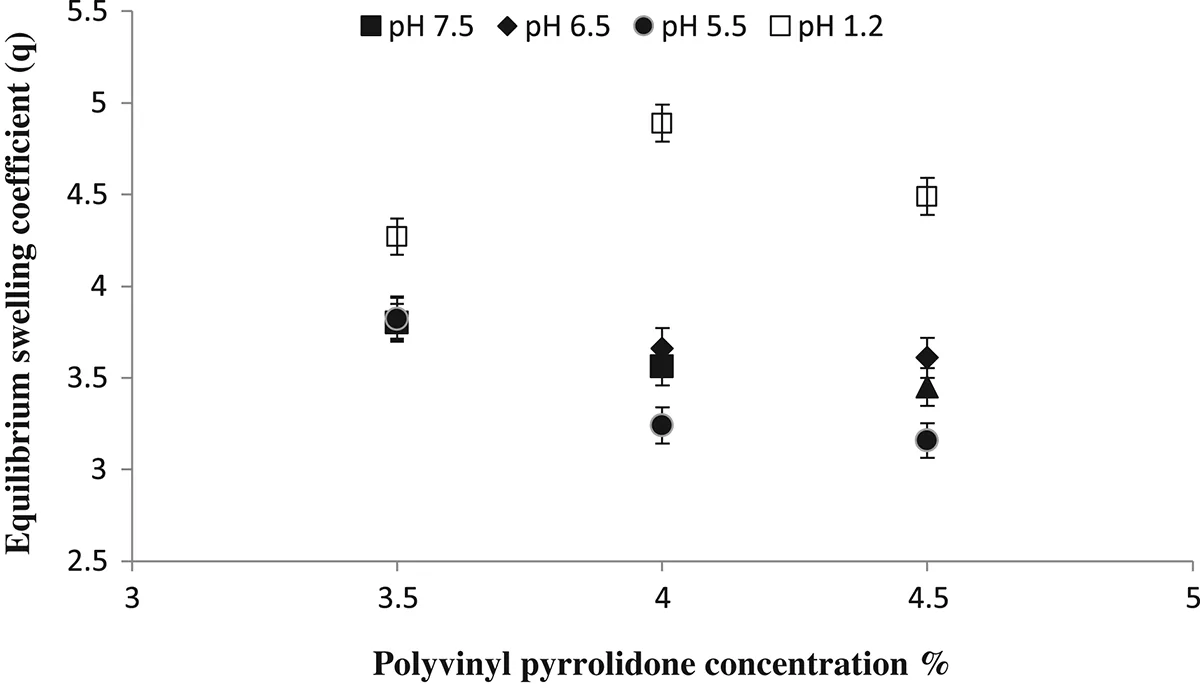
Equilibrium swelling ratio of Ge/PVP hydrogels having varying concentrations of PVP (3.5, 4 and 4.5 g) using GA as crosslinking agent (3.8 wt% of Ge and PVP) in solutions of different pH; pH 1.2 (□), pH 5.5 (●), pH 6.5 (♦) and pH 7.5 (▲).The data present the mean ± standard deviation of *n* = 3 individual readings.

### 3.4. Effect of the concentration of the crosslinking agent (GA) on swelling and drug release of Ge/PVP hydrogels

The increasing concentration of the crosslinking agent (GA) was also observed to investigate an effect on the drug release and swelling behavior of the hydrogel. To investigate the effect of GA on drug release and swelling behavior, three formulation of Ge/PVP hydrogel with different concentration of GA (3.5, 4.0 and 4.2wt % of Ge and PVP) were prepared as shown in Table 1. It was noted that the swelling behavior the hydrogel was inversely proportional to the GA concentration as shown in the Figure 7. The presence of more physical entanglements between the hydrogels with higher concentration of GA causes reduced swelling of the hydrogel. Increasing the concentration of GA can be correlated with decreasing the mesh size of the network. The process of ionization also reduces with higher concentration of GA. Reduced relaxation of the hydrogel network was observed with the higher crosslinking ratio that causes lower swelling of the hydrogel. Park et al. observed similar decrease in swelling of the hydrogel [40].

**Figure 7.**
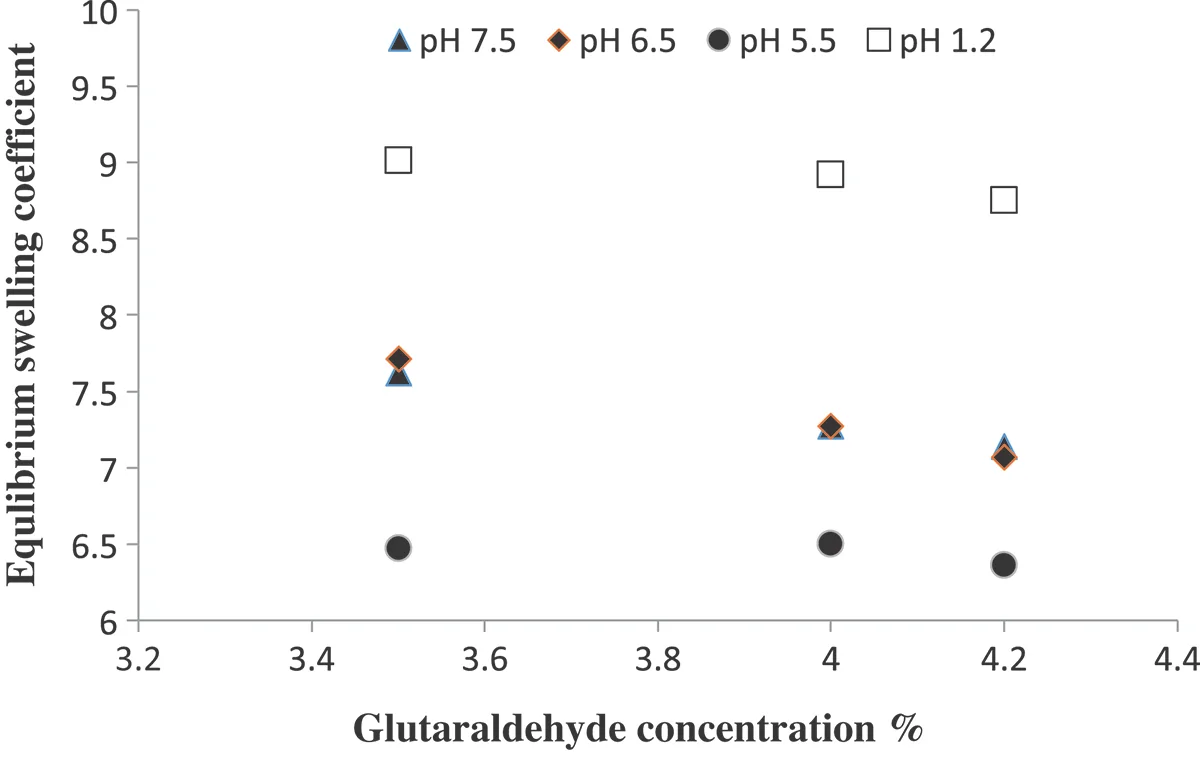
Equilibrium swelling ratio of Ge/PVP hydrogels having varying concentrations GA as crosslinking agent (3.5 wt%, 4 wt% and 4.2 wt% of Ge and PVP) in solutions of different pH; pH 1.2 (□), pH 5.5 (●), pH 6.5 (♦) and pH 7.5 (▲).

Release studies of drug in different buffer solution suggested that percent drug release was decreased with an increase in GA concentration owing to their swelling behavior as shown in the Figure 8(B).

**Figure 8.**
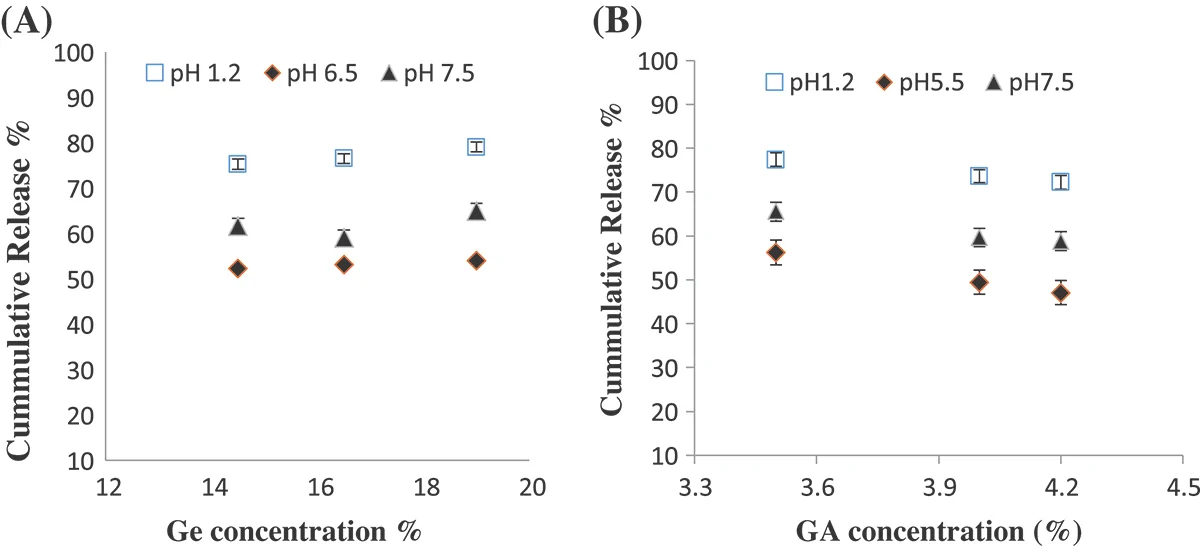
Cumulative % release of captopril from Ge/PVP hydrogels (A) Effect of variable concentration of Ge on % drug release in solution of different pH values (B) Effect of crosslinking agent concentration on % drug release in solution of different pH values. The data present the mean ± standard deviation of *n* = 3 individual readings.

### 3.5. Sol-gel fraction analysis

Sol-gel fraction was used to calculate the un-crosslinked polymer in the hydrogel. Sol-gel analysis revealed that Ge, PVP and GA concentration were directly proportional to the gel fraction as shown in the Table 4. On the other hand sol-fraction decreases with increase in the concentration of Ge, PVP and GA. The influence of both the polymers and GA concentration on the gel fraction of the hydrogel are shown in Figure 9.

**Table 4.** Gel fraction of different formulation of Ge/PVP hydrogels.

Sample codes	Ge/PVP ratio	Degree of crosslinking GA (wt%)	Gel fraction (%)
S_1_	82.85/17.15	3.8	80.21
S_2_	84.61/15.39	3.8	84.14
S_3_	86.36/13.46	3.8	84.77
S_4_	82.50/17.50	3.8	83.53
S_5_	80.48/19.52	3.8	83.77
S_6_	78.57/21.43	3.8	84.09
S_7_	84.61/15.39	3.5	82.79
S_8_	84.61/15.39	4.0	85.00
S_9_	84.61/15.39	4.2	88.63

**Figure 9.**
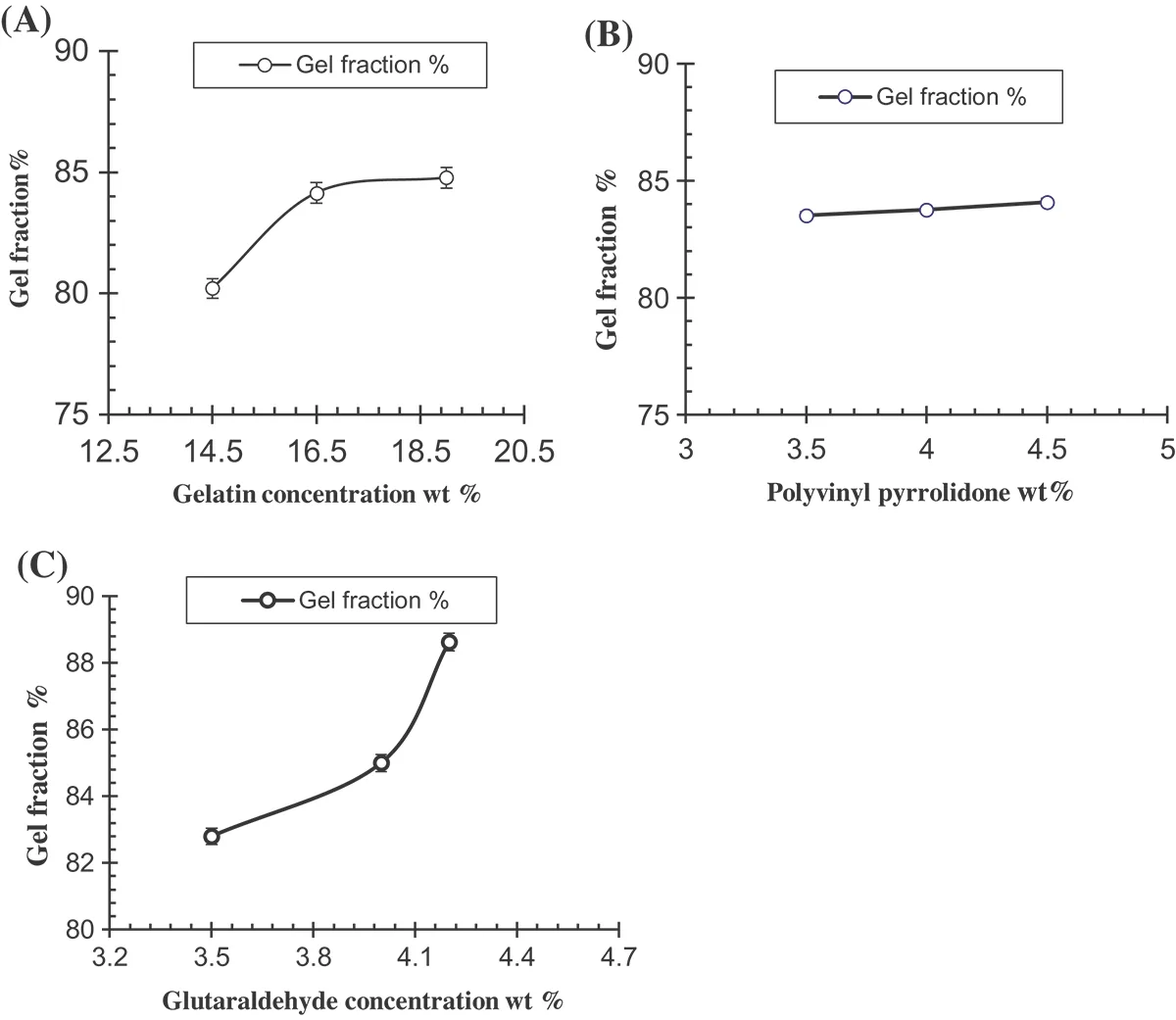
Effect of processing variables on gel fraction of Ge/PVP hydrogels (A) Ge Concentration (B) PVP Concentration (C) GA Concentration, The data present the mean ± standard deviation of *n* = 3 individual readings.

### 3.6. Porosity measurement

Analysis of the porosity measurement revealed that increasing the concentration of Ge and PVP causes increase in the porosity due to increase in the viscosity of the hydrogel solution. Sometimes the bubbles are prevented to escape from the solution that results in the formation of inter connected channels due to which porosity is increased. When the concentration of the crosslinking agent is increased, it results in increased entanglement between the monomers and polymers as a result porosity is decreased. Increase in the concentration of GA causes increase physical entanglement between Ge and PVP that results in decrease porosity as shown in Table 5. Figure 10 demonstrate the effect of polymer concentrations and GA concentration on porosity of the hydrogel.

**Table 5.** Porosity of different formulations of Ge/PVP hydrogels.

Sample codes	Ge/ PVP ratio	Degree of crosslinking GA (wt %)	Porosity (%)
S_1_	82.85/17.15	3.8	28.84
S_2_	84.61/15.39	3.8	34.99
S_3_	86.36/13.46	3.8	38.53
S_4_	82.50/17.50	3.8	18.11
S_5_	80.48/19.52	3.8	23.34
S_6_	78.57/21.43	3.8	25.74
S_7_	84.61/15.39	3.5	31.01
S_8_	84.61/15.39	4.0	26.22
S_9_	84.61/15.39	4.2	21.87

**Figure 10.**
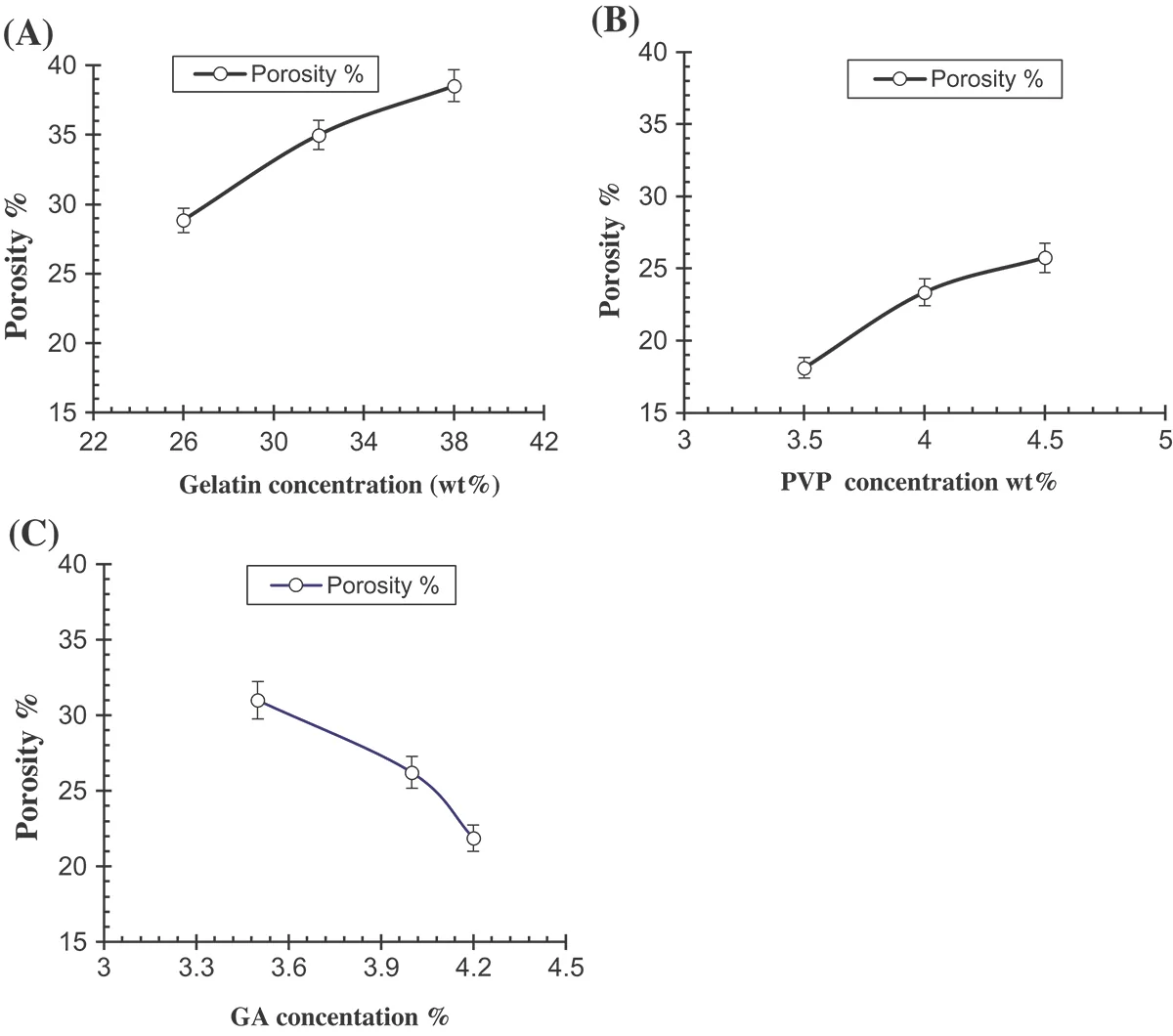
Effect of processing variables on porosity of Ge/PVP hydrogels (A) Ge concentration (B) PVP concentration (C) GA concentration. The data present the mean ± standard deviation of *n* = 3 individual readings.

### 3.7. Diffusion coefficient of polymers (D)

Fick’s first law of diffusion was employed in membrane diffusion or sorption and desorption technique. Diffusion coefficient was applied indirectly for the determination of diffusion of solute into the hydrogel. It was observed that with an increase in the concentration of GA, diffusion coefficient was also affected as shown in the Table 6.

**Table 6.** Flory-Huggins network parameters of Ge/PVP hydrogels.

Sample codes	V_2,*s*_	*Χ*	*M*_*c*_	*M*_*r*_	*q*	*D* (cm²/s)
S_1_	0.046433	−0.61261	5713.655	93.60281	59.82581	0.259520
S_2_	0.056222	−0.61237	6129.121	92.95433	64.59157	0.196683
S_3_	0.054342	−0.61169	6139.349	92.42910	65.00799	0.180310
S_4_	0.051952	−0.61433	4526.779	96.37461	46.97837	0.249044
S_5_	0.049411	−0.51359	4710.133	97.46823	48.45973	0.230588
S_6_	0.048882	−0.61341	5442.316	98.42065	55.59944	0.229931
S_7_	0.045461	−0.61217	5570.246	95.78332	57.22161	0.235711
S_8_	0.051231	−0.61433	4234.568	97.08011	42.98879	0.292192
S_9_	0.053444	−0.61490	4184.892	99.50739	42.03866	0.321289

#### 3.7.1. Solvent interaction parameters (χ) and molecular weight between crosslink (M_c_)

It was noted that the value of molecular weight between crosslink (*M*_*c*_) increased with increase in the concentration of Ge. Higher swelling of the polymer was observed due to the amino group of gelatin into the polymer chain. Average molecular weight between crosslinks and Ge values were also related to the cross-linked density as shown in the Table 6. Solvent interaction parameter (*χ*) between polymer and solvent was also studied. It was noted that higher the value of (*χ*), lower will be the values of interaction between polymer and solvent.

### 3.8. Mechanism of drug release

Phosphate buffer solution of different pH 1.2, 6.5, and 7.5 were used for the investigation of drug release pattern. The data obtained was evaluated for release pattern by fitting it into different models like Higuchi, Korsmeyer Peppas, zero order and first order as shown in Tables 7 and 9.

**Table 7.** Effect of Ge concentration on drug release kinetics of Ge/PVP hydrogels in different pH solutions using GA as crosslinker (3.8 wt% of Ge and Pec).

Sample codes	Ge contents (%)	pH	Zero order Kinetic		First order Kinetics		Higuchi *M*odel	
			*K*_0_ (*h* ^−1^)	*r*	*K*_1_ (*h* ^−1^)	*r*	*K*_2_ (*h* ^−1^)	*r*
S_1_	14.5	1.2	6.021	0.992	0.1175	0.987	0.257	0.979
		6.5	4.619	0.981	0.0877	0.990	0.201	0.970
		7.5	5.203	0.981	0.0697	0.992	0.225	0.971
S_2_	16.5	1.2	6.194	0.994	0.1207	0.961	0.261	0.953
		6.5	4.888	0.998	0.0689	0.987	0.198	0.958
		7.5	5.124	0.993	0.0791	0.985	0.209	0.963
S_3_	19.0	1.2	6.484	0.997	0.1251	0.968	0.265	0.966
		6.5	4.576	0.995	0.0654	0.986	0.183	0.957
		7.5	5.616	0.996	0.0912	0.988	0.226	0.971

**Table 8.** Effect of Ge concentration on drug release mechanism of Ge/PVP hydrogels in different pH solutions using GA as crosslinker (3.8 wt% of Ge and Pec).

Sample codes	Ge content (%)	pH	Release exponent (*n*)	*r*	Order of release
S_1_	14.5	1.2	0.695	0.989	Non-fickian
		6.5	0.906	0.994	Non-fickian
		7.5	0.751	0.982	Non-fickian
S_2_	16.5	1.2	0.709	0.976	Non-fickian
		6.5	0.999	0.996	Non-fickian
		7.5	0.742	0.988	Non-fickian
S_3_	19.0	1.2	0.732	0.989	Non-fickian
		6.5	0.671	0.966	Non-fickian
		7.5	0.726	0.985	Non-fickian

**Table 9.** Effect of concentration of crosslinker (GA) on drug release kinetics of Ge/PVP hydrogels in different pH solutions.

Sample codes	GA contents (%)	pH	Zero order kinetic		First order kinetics		Higuchi model	
			*K*_0_ (*h* ^−1^)	*r*	*K*_1_ (*h* ^−1^)	*r*	*K*_2_ (*h* ^−1^)	*r*
S_7_	3.5	1.2	6.321	0.985	0.1302	0.983	0.272	0.974
		6.5	5.251	0.989	0.0789	0.980	0.221	0.949
		7.5	5.629	0.973	0.1021	0.989	0.247	0.973
S_8_	4.0	1.2	6.019	0.994	0.1131	0.980	0.256	0.970
		6.5	4.811	0.988	0.0667	0.988	0.197	0.968
		7.5	5.158	0.996	0.0994	0.987	0.210	0.967
S_9_	4.2	1.2	6.045	0.984	0.1129	0.990	0.262	0.981
		6.5	4.818	0.988	0.0651	0.981	0.195	0.946
		7.5	5.402	0.995	0.0819	0.982	0.220	0.961

The selection of most suitable model for drug release was based on regression coefficient values (*r*) which defines the release of the drug from the device. Tables 8 and 10 show the regression coefficient (*r*) values from loaded hydrogels of Ge/PVP with varying concentration of Ge and crosslinker. Most of the samples showed greater regression coefficient (*r*) value for zero order release rate than first order. Thus it is concluded that the drug release from the samples having different concentration of Ge and crosslinker (GA) are according to the zero order release. The regression coefficient (*r*) value in Higuchi model revealed that the drug release followed the diffusion controlled mechanism.

**Table 10.** Effect of concentration of crosslinker (GA) on drug release mechanism of Ge/PVP hydrogels in different pH solutions.

Sample codes	GA contents (%)	pH	Release exponent (*n*)	*r*	Order of release
S_7_	3.5	1.2	0.706	0.991	Non-fickian
		6.5	0.993	0.994	Non-fickian
		7.5	0.776	0.989	Non-fickian
S_8_	4.0	1.2	0.739	0.989	Non-fickian
		6.5	0.998	0.895	Non-fickian
		7.5	0.778	0.991	Non-fickian
S_9_	4.2	1.2	0.798	0.997	Non-fickian
		6.5	0.999	0.997	Non-fickian
		7.5	0.917	0.995	Non-fickian

### 3.9. Instrumental analysis

#### 3.9.1. FTIR analysis

In order to assess the functional groups and confirm the chemical structure of cross-linked network from the hydrogels, samples were analyzed by Fourier transform infrared spectroscopy (FTIR). Figure 11 shows the FTIR spectra of pure Ge, pure PVP, crosslinked unloaded Ge/PVP hydrogel sample, loaded Ge/PVP hydrogel sample and pure drug.

**Figure 11.**
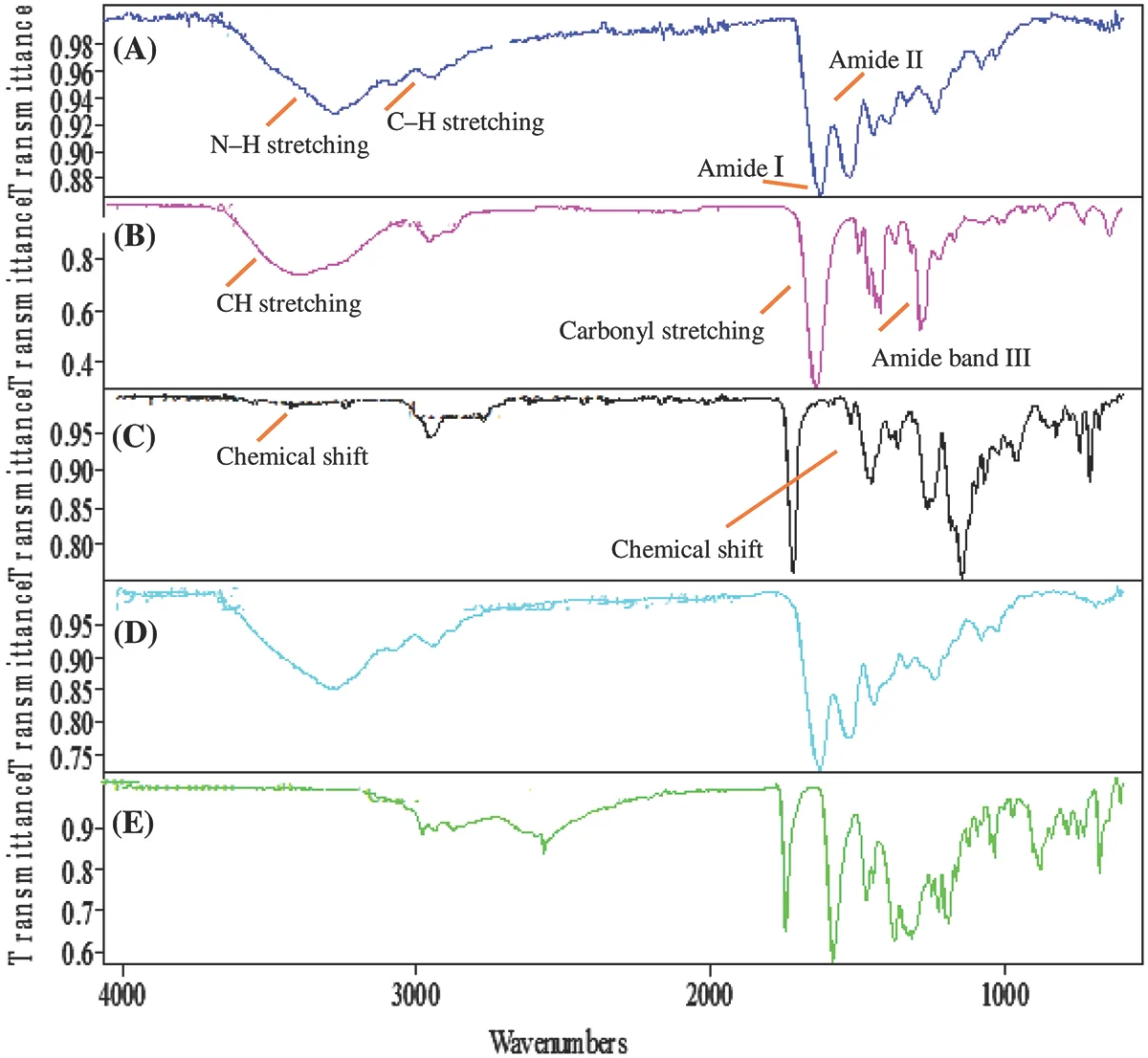
FT-IR spectra of (A) Gelatin (B) PVP (C) Unloaded hydrogel sample (D) Loaded hydrogel sample (E) Captopril.

In the FTIR spectra of gelatin as shown in Figure 11(A), the absorption peak at 3349 cm^−1^ is attributed to N–H stretching. Peaks at 3092 and 2998 cm^−1^ are attributed to C–H stretching. The peak at 1651 cm^−1^ refers to the absorption band of amide I and at 1564 cm^−1^ shows the absorption band of amide II. In the FTIR spectra of PVP shown in Figure 11(B), peaks at 2924 cm^−1^ attributed to CH stretching, a stretching peak between 1650 and 1659 cm^−1^ is attributed to carbonyl stretching (C=O) and 1290 cm^−1^ is attributed to amide band III (C–N stretch). In the FTIR spectrum of Ge/PVP sample shown in Figure 11(C), a shift in the bands was observed which shows the evidence of chemical interaction between the functional groups. In the FTIR spectrum of drug loaded Ge/PVP hydrogel sample shown in Figure 11(D), no significant changes were observed, which indicates that there is no chemical interaction between the drug and polymers. Figure 11(E) shows the FTIR spectra of captopril. From FTIR spectra of drug loaded sample it is clear that there is no chemical interaction refereeing the biocompatibility of drug in cross-linked gel network.

#### 3.9.2. XRD analysis

X-ray diffraction is a proven tool to provide very useful information on degree of sample crystallinity. The XRD pattern of Ge/PVP hydrogel and drug loaded Ge/PVP hydrogel has been shown in Figure 12. The diffract gram of unloaded Ge/PVP hydrogel indicated the major peak at ~43.360°, 51.180°, 57.460°, 64.780° and 75.840° at (2*i*). While the diffract gram of drug loaded Ge/PVP hydrogel indicated peaks at the same pattern with major peaks at ~ 44.940°, 53.360°, 59.410°, 69.260°, 78.220° at (2*θ*) as that of Ge/PVP hydrogel sample without drug. It means that there is no apparent interaction reported between drug and hydrogel.

**Figure 12.**
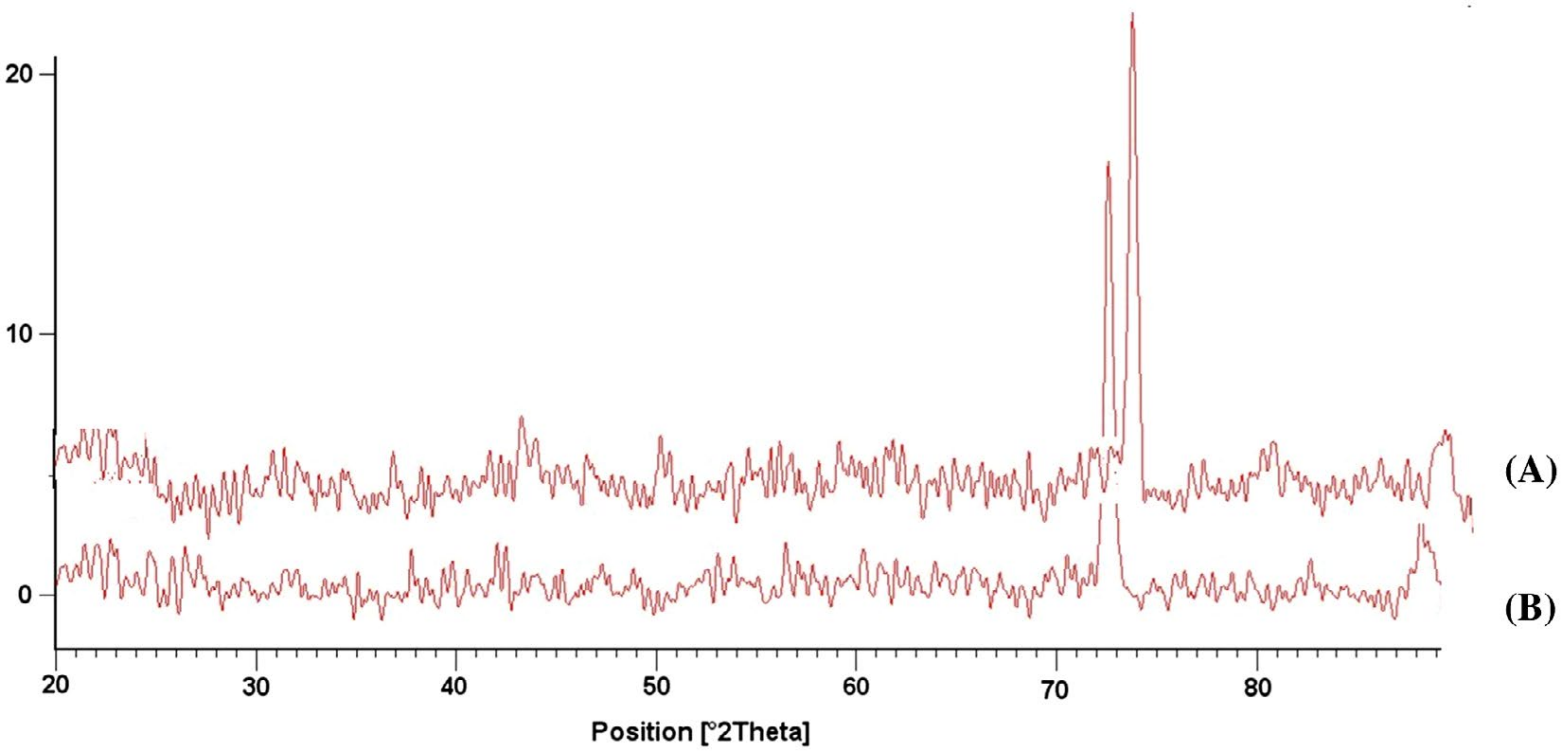
XRD spectra of (A) drug loaded Ge/PVP hydrogel sample (B) unloaded Ge/PVP hydrogel sample.

#### 3.9.3. SEM analysis

SEM analysis was performed to study the surface and cross-sectional morphology of drug loaded and unloaded Ge/PVP hydrogel sample. Figure 13 elaborate the surface phenomena and cross section of the unloaded and loaded hydrogel samples. The results shows the porous structure of the hydrogels which are assumed to act as channels for the adherence of the drug into the interpenetrating network of the hydrogel. This confirms the results obtained from porosity measurement of the hydrogels. Furthermore the cross-sectional SEM analysis of drug loaded sample confirmed the distribution of the drug within the matrix of the hydrogels as shown in Figure 13(E).

**Figure 13.**
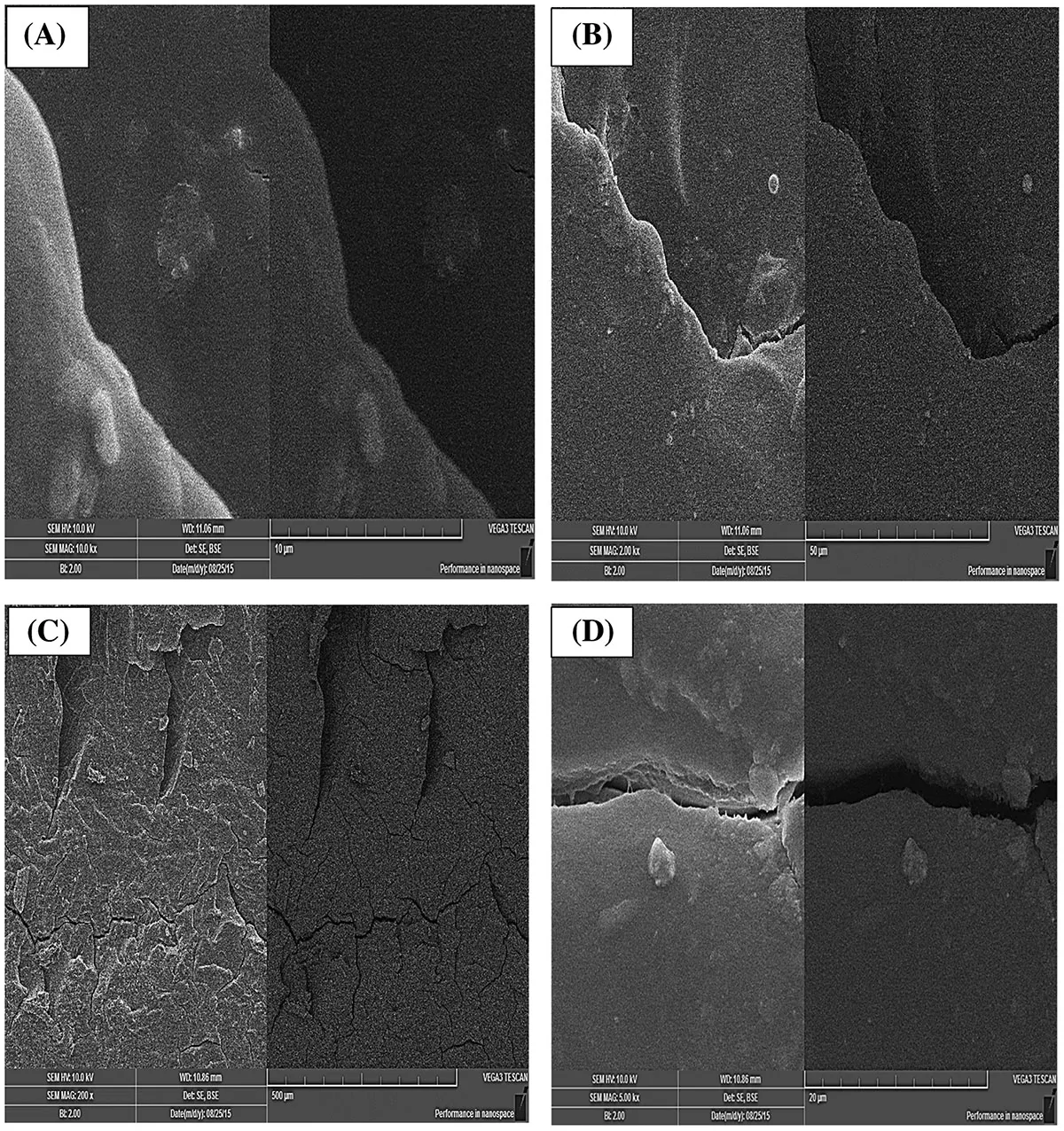
SEM analysis of Surface morphology of Ge/PVP hydrogel (A) Unloaded sample (B) loaded sample (C) Cross-sectional morphology of Unloaded Ge/PVP hydrogel sample (D) loaded Ge/PVP hydrogel sample.

## 4. Conclusion

Ge/PVP based pH sensitive hydrogels were synthesized in the present study employing GA as cross linker for the delivery of water soluble drugs. Composition and pH of the swelling medium greatly affect the swelling behavior of the prepared hydrogels. Buffer solutions of different pH i.e., 1.2, 5.5, 6.5 and 7.5 were used for the investigation of swelling behavior. Regular variation in the swelling ratio was observed with changing concentration of both the polymers and crosslinking agent. Increase in the concentration of Ge and PVP has direct relationship with swelling ratio while increase in GA concentration has inverse effect with the swelling ratio. Composition as well as pH of the swelling medium greatly affect the drug release from the hydrogel. Faster release of drug was observed at pH 1.2 in comparison with other pH solutions. It was concluded that these hydrogels showed pH sensitivity and are capable of showing response to the environmental conditions. So they may be used for the targeting of hydrophilic drugs at specific sites. Drug release from all the formulation showed non-Fickian mechanism.
